# A retrospective study: establishment and validation of the prediction model for the gonadotropin starting dose in IVF/ICSI-ET among normal ovarian response women

**DOI:** 10.3389/fendo.2025.1600936

**Published:** 2025-06-23

**Authors:** Yindi Zhang, Na Zhang

**Affiliations:** Department of Reproductive Medicine, The Fourth Hospital of Hebei Medical University, Shijiazhuang, China

**Keywords:** clinical prediction model, nomogram, IVF/ICSI-ET, Gn starting dose, NOR

## Abstract

**Purpose:**

This study aims to create and validate a clinical prediction model to determine the optimal gonadotropin (Gn) starting dose in controlled ovarian stimulation (COS) protocols for normal ovarian response (NOR) patients undergoing their first IVF/ICSI-ET cycle.

**Methods:**

A retrospective analysis was conducted based on the data of the first IVF/ICSI-ET cycles of 535 patients from the Reproductive Medicine Department of the Fourth Hospital of Hebei Medical University between January 2017 and June 2024. The patients were randomly divided into a training group (n=317) and a validation group (n=218) in a 6:4 ratio. Linear regression analysis was applied to screen out the potential factors influencing the Gn starting dose, and the statistically significant factors were selected to construct a nomogram for Gn dosage. We used an internal verification method to ensure the reliability of the nomogram.

**Results:**

The patient’s age, body mass index (BMI), basal follicle-stimulating hormone (bFSH), antral follicle count (AFC), and anti-Müllerian hormone (AMH) were predictive indicators of the Gn starting dose for NOR patients undergoing IVF/ICSI-ET treatment (*P*<0.05). A predictive model was created based on the above indicators. Finally, the accuracy of this predictive model was validated by comparing the actual Gn starting doses with the predicted doses in both the training and the validation group. The results showed no significant difference between the actual and predicted Gn starting doses in the two groups (*P*>0.05).

**Conclusion:**

Based on age, BMI, bFSH, AMH, and AFC, a clinician could determine the patient’s appropriate Gn starting dose for NOR patients undergoing IVF/ICSI-ET.

## 1 Introduction

*In vitro* fertilization and intracytoplasmic sperm injection-embryo transfer (IVF/ICSI-ET) are the most widely used assisted reproductive technologies (ART), enabling millions of infertile couples to achieve pregnancy by overcoming gamete transport, fertilization, or developmental barriers. Normal ovarian response (NOR) is defined as the recruitment of 5–15 mature follicles following controlled ovarian stimulation (COS), representing 70%–90% of ART cycles worldwide. This population serves as the clinical backbone of IVF, yet personalized dosing strategies for NOR remain underdeveloped compared to extreme responders (1). In recent years, “one size fits all” concept in IVF had gradually evolved into the “individualization” (2). There are multiple aspects of assisted IVF treatment that can be individualized to optimize treatment outcomes, including COS, ovulation triggering and luteal phase support (3). The use of Gn for COS is a core element for the success of IVF, achieved through daily injections of exogenous FSH to maintain FSH concentrations above the threshold for single follicle development for several days, thereby promoting the maturation of multiple follicles and resulting in the retrieval of multiple oocytes (4).

According to the ESHRE guidelines for ovarian stimulation during IVF treatment, although it remained unclear whether this individualized approach had been beneficial in terms of the live birth rate, dose individualization in patients could minimize the risks of ovarian hyperstimulation syndrome (OHSS), iatrogenic poor ovarian response, and cycle cancellation (5–7). Therefore, the application of individualized COS in IVF is important. Despite the fact that exogenous FSH has been used for decades and millions of cycles have been carried out globally to date, the ovarian response often remains unpredictable. Therefore, individualized stimulation protocols, including drug combinations, dosages, and adjuvant drugs, have not been clearly identified. The individualized adjustment of ovarian stimulation protocols based on FSH starting doses still lacks a unified standard (8). Clinicians often rely on empirical judgment rather than data-driven models, highlighting the need for standardized, evidence-based dosing tools. To breakthrough the limitations of clinical decision-making in the current individualized COS protocols, researchers have developed several predictive models. In 2006, Howles et al. (9) first constructed a multifactorial predictive model that included bFSH, BMI, age, and AFC, with a concordance index of 59.5%. It marked the beginning of quantitative research on Gn dose prediction. Although subsequent studies have continuously expanded the predictive dimensions, there are often limitations in variable selection. The models either incorporate only single indicators such as age and follicle-stimulating hormone (FSH), or overlook key biomarkers like body mass index (BMI), antral follicle count (AFC), and anti-Müllerian hormone (AMH) (10). Popovic-Todorovic et al. developed a scoring system for calculating the FSH starting dose, based on the total number of antral follicles, total Doppler score, serum testosterone concentrations and smoking habit (11). However the predictive model exhibits notable limitations in its selection of indicators, as it didn’t include critical parameters such as patient age and AMH, which are essential for assessing ovarian reserve function. This omission significantly restricts the clinical applicability and predictive accuracy of the model. La Marca et al. constructed a nomogram based on age, AMH, and bFSH, emphasizing that age and AMH are the most important predictive factors (12). Following this, Moon et al. further validated the predictive efficacy of this biomarker combination using a generalized linear model (13). These advancements have provided new theoretical support for the optimization of the Gn dose model.

The predictive models previously discussed exhibit several limitations regarding their predictive indicators and primarily targeting populations characterized by poor ovarian response (POR) or high ovarian response (HOR). However, there is a lack of relevant research on the NOR population, which represents the majority of individuals undergoing ART treatment, accounting for approximately 70% to 90% of cases. Therefore, there is an urgent need to develop a Gn dosing prediction model specifically for the NOR population, incorporating key biomarkers. This study aims to fill this gap.

## 2 Materials and methods

### 2.1 Patients’ selection

For this retrospective study, we initially screened 1098 patient records from the hospital database between January 2017 and June 2024 for potential inclusion. Finally, according to the inclusion and exclusion criteria, a total of 535 women with normal ovarian response (NOR) undergoing their first IVF/ICSI-ET cycles at the Reproductive Medicine Center of the Fourth Hospital of Hebei Medical University were enrolled in the study. The inclusion criteria were as follows: 1. Patients who received their first IVF/ICSI-ET treatment with gonadotropin-releasing hormone agonist (GnRH-a) or gonadotropin-releasing hormone antagonist (GnRH-A) protocol; 2. Patients aged between 20 to 38 years; 3. Patients with a regular menstrual cycle (28 ± 7 days). 4. Patients whom the number of retrieved oocytes ranged between 5 and 15. The exclusion criteria were as follows: 1. Patients diagnosed with endocrine diseases, metabolic diseases and autoimmune diseases, such as hyperprolactinemia, polycystic ovary syndrome, systemic lupus erythematosus; 2. Patients had chromosomal abnormalities.

### 2.2 Controlled ovulation stimulation protocol

In this study, a total of 326 patients underwent COS using the long-acting GnRH agonist (GnRH-a) protocol. On the second or third day of menstruation, a long-acting GnRH-a was administered to patients for pituitary downregulation. Twenty-eight days later, these patients started COS treatment with exogenous Gn. Clinician adjusted the dosage of Gn according to the follicle size and hormone levels. When more than two follicles reach a diameter of ≥18 mm, these patients were given an injection of hCG trigger, and oocyte retrieval via vaginal vault puncture is carried out 36 hours after triggering.

A total of 209 patients underwent COS using the GnRH antagonist (GnRH-A) protocol. On the the second or third day of menstruation, these patients started COS treatment with exogenous Gn. When the largest follicle reached a diameter of 12–14 mm or the E_2_ level reached 400 pg/ml, GnRH-A was added and continued until the ovulation trigger. The timing of trigger and oocyte retrieval was the same as GnRH-a protocol.

### 2.3 Data collection

Patient clinical parameters, including age, body height, body weight, BMI, body surface area (BSA), infertility duration, infertility type, infertility factors, history of pelvic surgery and abortus were recorded. The levels of basal estrogen (bE_2_), basal FSH (bFSH), basal luteinizing hormone (bLH), basal progesterone (bP), prolactin (PRL), AMH, and testosterone (T), as well as E_2_, P, LH levels, endometrial thickness on the trigger day and AFC were also measured. The initial and total doses of Gn, the duration of Gn were also recorded. All hormone concentrations were retrospectively extracted from electronic medical records, with basal levels (E_2_, FSH, LH, P, PRL, T) measured on day 3 of the menstrual cycle and mid-cycle levels (E_2_, P) on the day of hCG administration.

### 2.4 Statistical analysis

In accordance with the random sampling technique, the infertile couples were divided into training set and validation set at a ratio of 6:4. The continuous variables were represented as the mean ± standard deviation (SD), while non-normally distributed data were presented as the median (interquartile range). To compare variables between groups, Student’s t-test was applied for normally distributed data, and the Mann-Whitney U-test was utilized for non-normally distributed data. The categorical variables were expressed as percentages, and the chi-squared test was used for statistical comparison. Univariate and multivariate linear regression analyses were employed to identify predictive factors associated with the Gn starting dose. Moreover, all categorical variables were transformed into dummy variables before performing univariate analysis. The above data were analyzed and processed using IBM SPSS Statistics for Windows (version 27.0).

To intuitively present the comprehensive impact of various predictive factors on the Gn starting dose in the multifactor analysis, this study utilized R (version 4.3.1) to construct and plot a nomogram. To assess the accuracy of the model, we calculated the MAE, REMS and R^2^, as well as a t-test was conducted to compare the actual Gn starting dose with the model-predicted Gn starting dose in both the training group and the validation set. The *P*<0.05 was considered statistically significant.

## 3 Results

### 3.1 Baseline characteristics

The study included a total of 535 patients, who were divided into a training set (n=317) and a validation set (n=218) in a ratio of 6:4. Baseline characteristics of both groups are summarized in **Table 1**. As shown in **Table 1**, there were no statistically significant differences (P>0.05) in almost all characteristics between the two groups at baseline.

**Table 1:** Patient and IVF cycle characteristics of validation set and training set.

Characteristics	Training set (n=317)	Validation set (n=218)	*P*-Value
Age	32 [29;35]	32 [30;35]	0.055
Infertility duration	3 [1;4]	3 [2;6]	0.063
Infertility type			0.560
Primary infertility	48.90% (155/317)	46.30% (101/218)	
Secondary infertility	51.10% (162/317)	53.70% (117/218)	
Pelvic inflammation and tubal disease			0.061
Yes	52.37% (116/317)	60.55% (132/218)	
No	47.63% (151/317)	39.45% (86/218)	
Endometriosis adenomyosis			0.137
Yes	10.10% (32/317)	6.40% (14/218)	
No	89.90% (285/317)	93.60% (204/218)	
History of pelvic surgery			0.250
Yes	26.20% (83/317)	30.70% (67/218)	
No	73.80% (234/317)	69.30% (151/218)	
History of previous abortus			0.502
Yes	30.30% (96/317)	33.00% (72/218)	
No	69.70% (221/317)	67.00% (146/218)	
Body Height (cm)	160 [158;165]	161 [158;165]	0.739
Body Weight (kg)	60 [54;67]	60 [55;70]	0.439
BMI (Kg/m^2^)	22.90 [20.6;26]	23.40 [21.12;26.30]	0.443
BSA (m^2^)	1.72 [1.64;1.82]	1.72 [1.65;1.87]	0.282
AFC (n)	12 [9;15]	12 [9;16]	0.089
Basal E_2_ (pg/ml)	41.91 [33;55.57]	40 [32;53.98]	0.722
Basal LH (IU/L)	4.82 [3.32;6.4]	4.38 [3.47;5.6]	0.204
Basal FSH (IU/L)	7.06 [5.65;8.29]	6.79 [5.89;8.26]	0.837
Basal P (ng/ml)	0.29 [0.19;0.44]	0.27 [0.18;0.39]	0.869
PRL (ng/ml)	14.74 [11.23;18.07]	14.58 [11.10;18.08]	0.837
AMH (ng/ml)	2.08 [1.37;3.25]	1.89 [1.35;3.08]	0.394
T (ng/L)	0.21 [0.14;0.34]	0.20 [0.14;0.28]	0.135
COS protocol			0.158
GnRH-A (%)	36.60% (116/317)	42.70% (93/218)	
GnRH-a (%)	63.40% (201/317)	57.30% (125/218)	
Fertilization			0.552
IVF (%)	84.90% (269/317)	86.70% (189/218)	
ICSI (%)	15.10% (48/317)	13.30% (29/218)	
Gn starting dose (IU/day)	225 [200;300]	225 [200;300]	0.367
Total Gn dose (IU)	2600 [1962.5;3500]	2700 [2075;3400]	0.593
Duration of stimulation (days)	11 [9;13]	10 [9;12]	0.946
E_2_ on the trigger day (pg/ml)	2258 [1640;2923]	2482 [1718;3000]	0.071
LH on the trigger day (pg/ml)	1.47 [0.9;2.61]	1.85 [0.9;3.45]	0.451
P on the trigger day (pg/ml)	0.70 [0.52;0.97]	0.68 [0.45;0.93]	0.605
Em on the trigger day (mm)	12.5521 ± 0.16075	12.5087 ± 0.18.338	0.904
Number of oocytes retrieved (n)	10 [7;12]	9 [7;13]	0.953

Continuous variables are shown as the median (interquartile range) or mean ± standard deviation. Categorical variables are presented as percent.Student’s t-test (for normally distributed data) or the Mann–Whitney U-test (for non-normally distributed data) were employed. Categorical variables were expressed as percentages, and the chi-squared test was used for statistical comparison.

BMI, body mass index; BSA, body surface area; AFC, antral follicle count; E2, estradiol; LH, luteinizing hormone; FSH, follicle-stimulating hormone; P, progesterone; PRL, prolactin; AMH, anti-Müllerian hormone and testosterone (T). Training set vs. validation set: *P*<0.05.

### 3.2 The correlation analysis between Gn starting dose groups and oocyte retrieval

**Figure 1:**
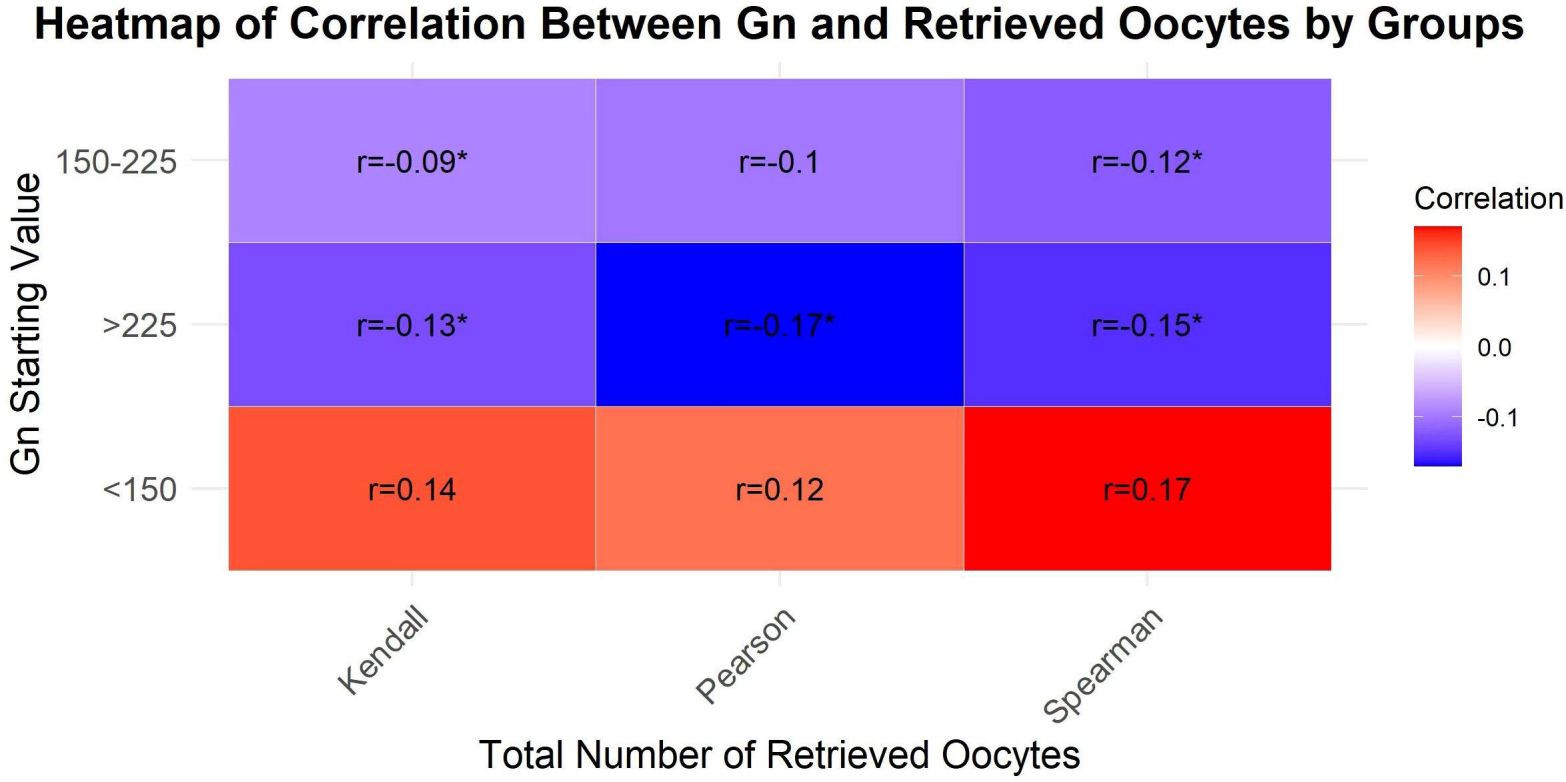
A heatmap to visualize the correlation coefficients across different groups and total number of retrieved oocytes.

The correlation analysis between Gn starting dose and oocyte retrieval results were showed in **Figure 1**. When the Gn starting dose was below 225 IU, there was no statistically significant correlation between the Gn starting dose and the number of retrieved oocytes (*P*>0.05). However, when the Gn starting dose exceeded 225 IU, a statistically significant negative correlation was observed between the Gn starting dose and the number of oocytes retrieved (*P*<0.05).

### 3.3 Linear regression analysis

**Table 2** presents the results of the univariate and multivariate linear regression analysis for Gn starting dose. As demonstrated, patients’ age, BMI and bFSH, were identified as positively correlated independent influencing factors (*P*<0.05). In contrast, AFC, bLH and AMH were found to be negatively correlated independent factors (*P*<0.05). Compared to primary infertility, secondary infertility did not have a significant effect on the Gn starting dose (*P*>0.05). Similarly, a history of pelvic surgery and induced abortion did not significantly affect the Gn starting dose (*P*>0.05). When statistically significant predictive indicators were included in the multivariate linear regression analysis, the results indicated that bLH (*P*>0.05) was no longer a predictive factor for the Gn starting dose.

**Table 2:** Predictors of the Gn starting dose in univariate and multivariate liner regression analysis.

Variables	Univariate analysis			Multivariate analysis		
	Regression coefficient	Standard error	*P*-Value	Regression coefficient	Standard error	*P*-Value
Age	4.356	0.251	<0.01	2.653	0.157	<0.01
Infertility duration	1.102	0.051	0.248			
Secondary infertility	-1.908	-0.015	0.721			
History of pelvic surgery (Yes)	7.701	0.056	0.194			
History of previous abortus (Yes)	1.424	0.011	0.804			
BMI (Kg/m^2^)	1.921	0.131	0.003	1.442	0.099	0.009
BSA (m^2^)	36.040	0.091	0.109			
AFC (n)	-4.172	-0.356	0.01	-2.236	-0.196	<0.01
Basal E_2_ (pg/ml)	0.133	0.055	0.205			
Basal FSH (IU/L)	5.321	0.183	<0.01	4.090	0.144	0.001
Basal LH (IU/L)	-2.509	-0.089	0.04	-2.074	-0.075	0.084
Basal P (ng/ml)	-9.794	-0.058	0.188			
PRL (ng/ml)	-0.054	-0.005	0.915			
AMH (ng/ml)	-19.126	-0.477	<0.01	-13.219	-0.339	<0.01
T (ng/L)	-1.454	-0.046	0.299			

### 3.4 Construct the nomogram and evaluate its accuracy.

**Figure 2:**
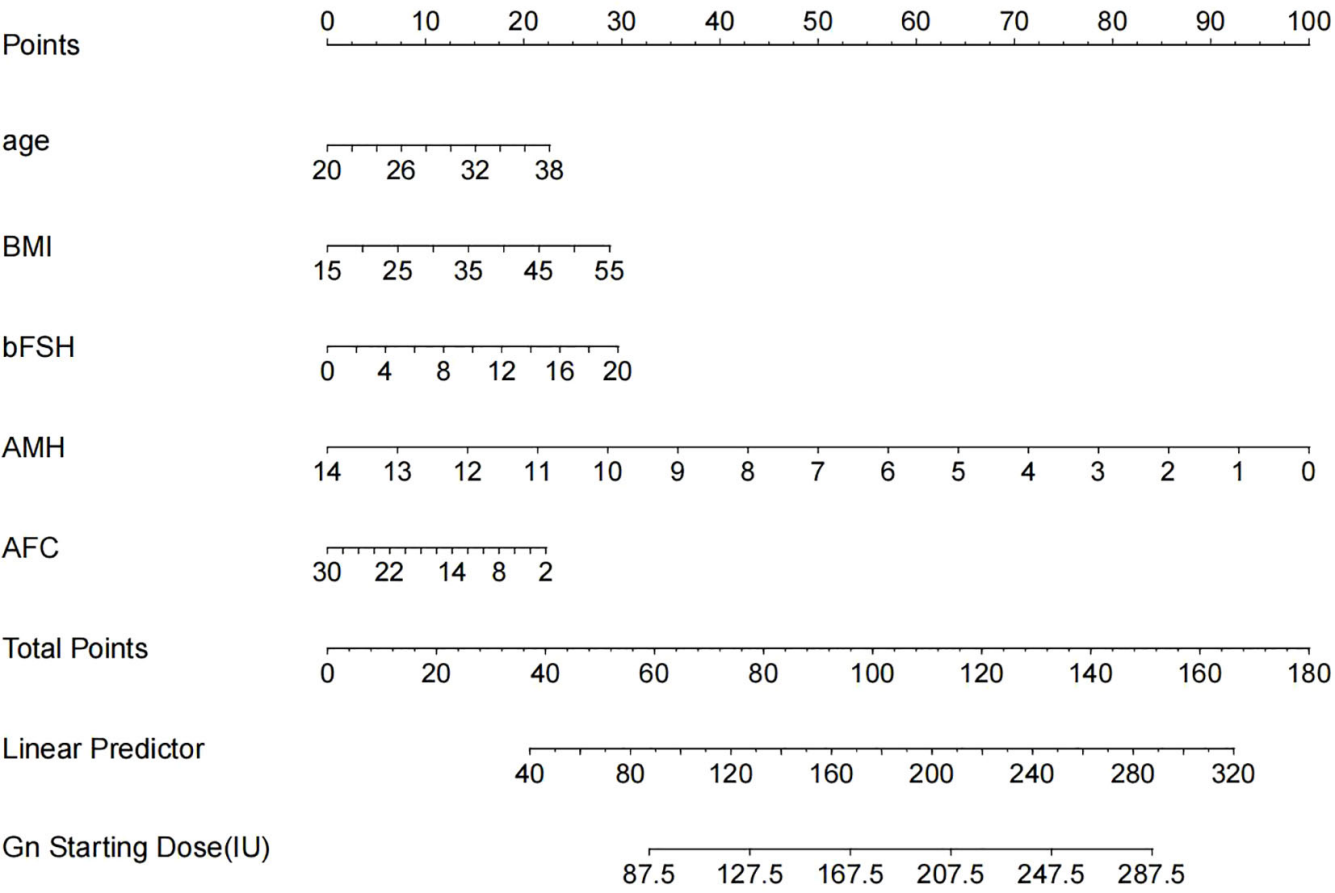
The nomogram is used to predict the gonadotrophin starting dose based on age, BMI, bFSH, AMH and AFC. The nomogram can be applied by following procedures: draw a line perpendicular from the corresponding axis of each physiological indicator until it reaches the top line labeled “Points”; sum up the points for all risk factors and recorded as the total score; and draw a line descending from the axis labeled “Total points” until it intercepts the lower line to determine the final value of Gn starting dose.

The result of the multiple regression analysis indicated that age, BMI, bFSH, AMH, and AFC were predictive factors for the Gn starting dose. Based on these findings, a predictive model was developed using these five predictors to calculate the Gn starting dose, and the model was visualized through a nomogram (**Figure 2**) created using R (version 4.3.1).

**Figure 3:**
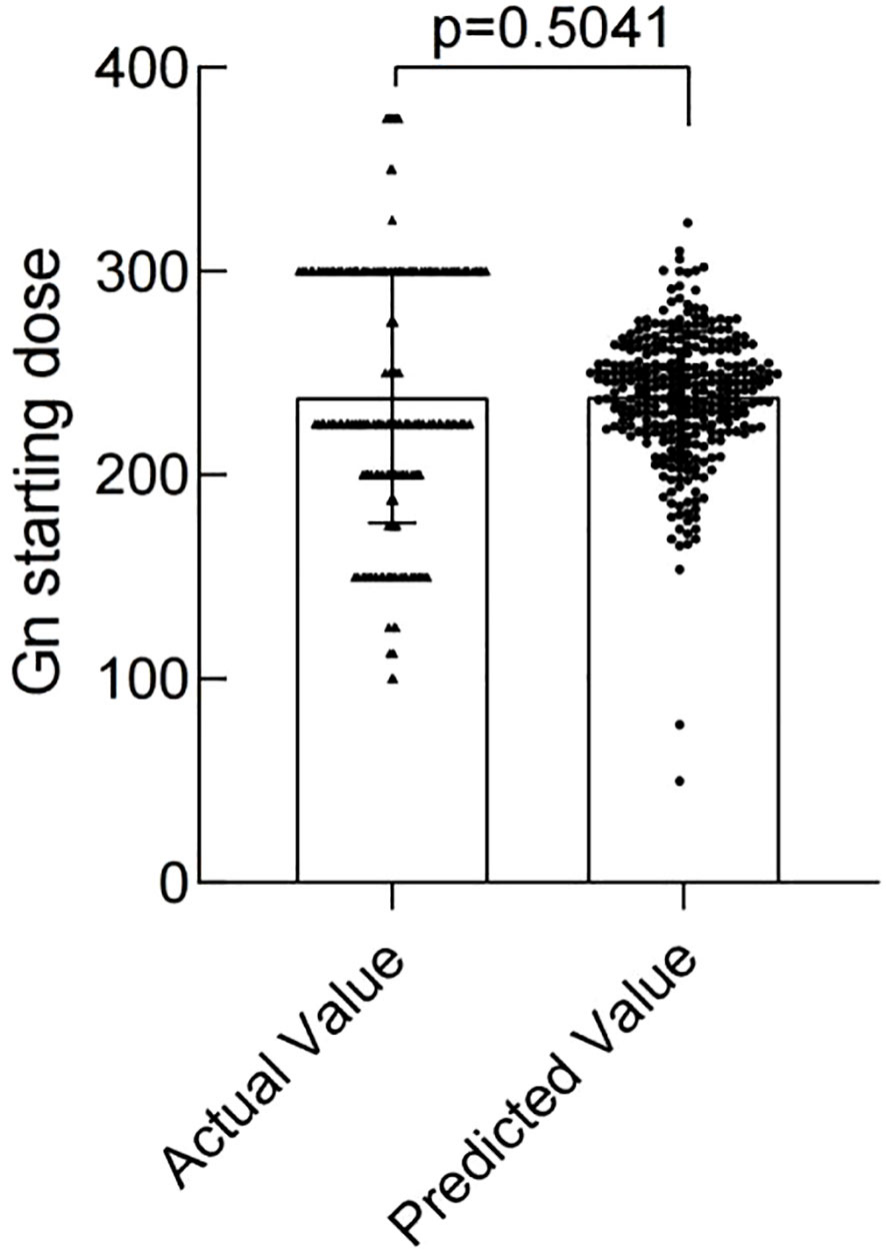
Comparison of Actual vs. Predicted Gn starting doses in Training Set.

**Figure 4:**
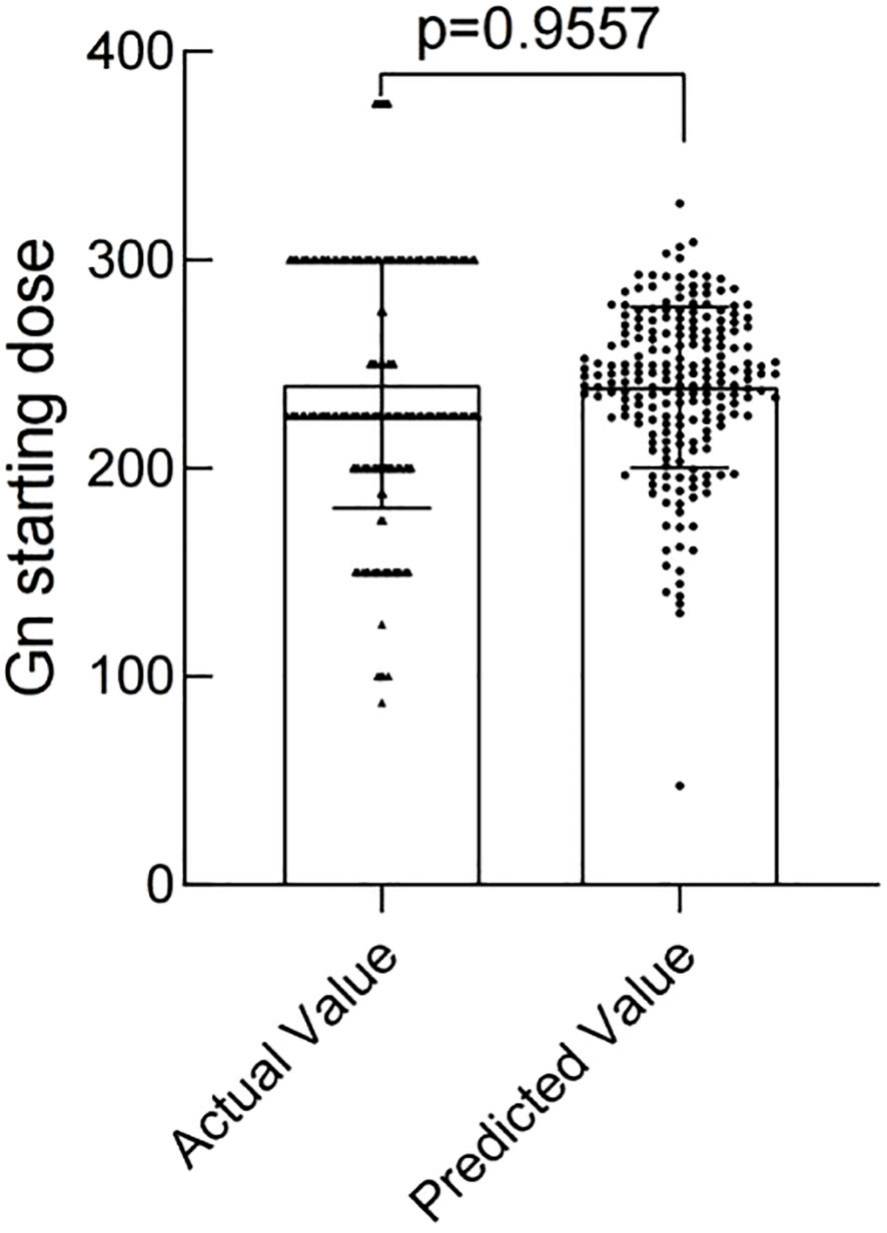
Comparison of Actual vs. Predicted Gn starting doses in Validation Set.

The modeling performance of the Gn starting dose model is shown in **Figure 3** and listed in **Table 3**. As shown in **Figures 3** and **4**, there were no statistically significant differences observed between the two groups (*P*>0.05), indicating that the prediction model demonstrates satisfactory predictive efficacy. The RMSE of the training and validation set were 50.94 and 50.71, respectively, and the MAE of the training and validation set were 40.90 and 39.76, respectively, with their R^2^ being 0.32 and 0.28, respectively.

**Table 3:** Training and validation set of predictive accuracy of the nomogram.

Dataset	RMSE	MAE	R^2^
Training set	50.94	40.90	0.32
Validation set	50.71	39.76	0.28

## 3 Discussion

With the rapid advancement of the IVF/ICSI-ET technology, there is an increasing focus on the personalization of diagnostic and therapeutic strategies. This ongoing optimization of processes and enhancement of precision significantly improved pregnancy rates among individuals experiencing infertility. Controlled ovarian stimulation (COS) plays an important role in IVF/ICSI-ET. COS involves the administration of exogenous Gn to stimulate the growth and development of multiple ovarian follicles, thereby facilitating the retrieval of multiple oocytes. However, determining the suitable Gn starting dose is still a challenge for clinicians.

Data in China showed that between 70% and 90% of individuals with NOR undergoing IVF/ICSI-ET. Therefore, the study aimed to develop a predictive model for individualized Gn starting dose tailored to NOR patients undergoing IVF/ICSI-ET treatment for the first time.

In this study, an analysis of the correlation between various Gn starting dose groups and the number of oocytes retrieved indicated that the retrieval of oocytes does not increase indefinitely with escalating Gn starting dose in the NOR patients. Notably, when the Gn starting dose exceeded 225 IU, a statistically significant negative correlation was observed between the starting dose and the number of oocytes retrieved. This finding aligns with prior research.

A machine learning model (14) based on the personalized Gn starting dose during the COS process showed that a nonlinear relationship existed between the number of MII oocytes and the FSH starting dose. Increasing FSH dosage raises MII oocyte numbers to a peak, after which they may plateau or decline. This phenomenon aligns with prior research, revealing excessive FSH can lead to a reduction in the number of oocytes retrieved (4). This conclusion is consistent with our research findings, which showed that within the Gn starting dose range of 150–225 IU, there was a negative correlation between the dose and the number of oocytes retrieved in NOR patients, but this association did not have statistical significance. In addition, a study on cattle (15) proved that there was a plateau phase in the maximal response to superovulation and excessive FSH doses had an adverse effect on the quality of oocytes and embryos. A plausible explanation for this phenomenon was that patients receiving high doses of Gn to induce ovulation may experience an earlier trigger due to the rapid increase E_2_ concentrations (14). A meta-analysis (16) revealed that increasing the Gn starting dose could result in a greater number of oocytes retrieved in patients with both POR and NOR. In our research findings, when the Gn starting dose less than 150 IU, the number of oocytes retrieved was positively correlated with the Gn starting dose, although this correlation did not reach statistical significance.

This study included five potential factors that may influence the Gn starting dose, including the patient’s age, BMI, bFSH, AFC, AMH. Utilizing these factors, a predictive model was developed to aid clinicians to calculate the optimal Gn starting dose. The accuracy of this predictive model was validated which showed that this predictive model can reliably forecast the Gn starting dose for NOR patients undergoing COS protocols. However, the value of R^2^ was not satisfactory, which might be caused by the limited sample size.

Age is a critical determinant to assessed ovarian reserve function, with age-related decline in fertility mainly manifested as a decreased quantity and quality of follicles and a declining ovarian response to exogenous Gn. In our study, multivariate linear regression analysis identified age as a persistent determinant influencing the Gn starting dose. However, a retrospective cohort study (17) showed that age ceased to be a significant independent variable for FSH starting dose adjustment in PCOS patients aged 20–40 years. A plausible explanation was that PCOS patients exhibit elevated ovarian reserve, rendering age statistically insignificant compared to other biomarkers in multivariate regression models. Similarly, Wu et al. (18) established a predictive model for Gn starting dose for POR patients, revealing no significant correlation between age and Gn starting dose.

It is common knowledge that weight and BMI are important risk factors associated with menstrual dysfunction and anovulation. A large-scale population-based study (19) indicated that both overweight and underweight women could significantly reduce the probability of conception and increase the risk of infertility and miscarriage. Previous studies (20) have demonstrated that obesity may adversely affect oocyte quality, embryo implantation, and embryonic development through pathophysiological mechanisms such as endocrine dysregulation, chronic inflammation, and ovarian dysfunction. In 2018, a meta-analysis (21) showed that overweight and obese women undergoing ART required a higher Gn starting dose. This finding suggested that obesity significantly reduced ART efficacy, which was consistent with the results of our study.

BSA, a key parameter in drug dosage calculation, more closely correlates with pharmacokinetics and pharmacodynamics than weight and age, reflecting interindividual physiological variations. However, our univariate analysis showed no significant association between BSA and Gn starting dose. La Marca et al. (12) developed a nomogram based on age, AMH and bFSH to predict the optimal Gn starting dose, but did not incorporate BMI and BSA.

Selecting appropriate ovarian reserve markers is fundamental for predicting the optimal ovarian response. The ESHRE (7) recommend using AFC or AMH levels to predict the responsiveness of the ovary to exogenous Gn stimulation. AFC is a key marker of ovarian reserve, defined as the number of antral follicles in the ovaries with a diameter of approximately 2-10mm determined by transvaginal ultrasonography in the early follicular phase. AMH is produced by the granulosa cells of early developing follicles, and its quantity can reflect the number of primordial follicles in the follicle pool (22). AFC is positively correlated with the number of recruited follicles (23), further highlighting its importance as an indicator of ovarian reserve. AMH is a reliable marker of ovarian reserve owing to its menstrual cycle stability and lower cycle-to-cycle variability compared with FSH (24, 25). An animal study (26) showed that AMH gene knockout in mice reduced ovarian reserve, accelerating follicular activation and increasing AFC. These findings indicate that AMH levels may more accurately reflect ovarian reserve than AFC.

Besides incorporating the above-mentioned factors, this study analyzed the effects of infertility type, history of pelvic surgery, and induced abortion on the Gn starting dose. Previous studies have suggested that ovarian surgery might impair the ovarian blood supply, thereby influencing the pharmacodynamics of Gn (6). However, our study revealed no significant association between a history of pelvic surgery or abortion and the Gn starting dose. Similarly, no significant statistical difference was observed between primary and secondary infertility in Gn dosing. It should be noted that the relevant data obtained in this study have certain limitations. Because in the overall sample data, the number of patients with a history of pelvic surgery was extremely small, which made the research on this specific group lack sufficient representativeness and comprehensiveness, thus affecting the accuracy and universality of the research results.

This is the first predictive model for the Gn starting dose tailored for individuals with NOR. Although the prediction model demonstrates clinical utility, several limitations should be noted. Due to the retrospective nature of our dataset, certain variables, including pregnancy-related indicators and male factors were not available for analysis. This lack of information may have introduced some degree of bias and could potentially affect the comprehensiveness of our model. Despite these limitations, our findings provide a foundation for further investigation. Future prospective studies should consider including pregnancy-related indicators and male factors to explore its potential impact on Gn starting dose more comprehensively. Longitudinal data collection with a wider range of variables could help refine the prediction model and improve its accuracy, thereby contributing to more personalized and effective clinical practice.

## 4 Conclusions

The patients’ age, BMI, bFSH, AMH and AFC are predictive indicators for the initial Gn dose in COS treatment, and the clinical prediction model constructed in this study can accurately predict the Gn starting dose in IVF/ICSI-ET treatment for NOR.

## Data Availability

The raw data supporting the conclusions of this article will be made available by the authors, without undue reservation.
